# Molecular and Immunological Characterization of Ragweed (*Ambrosia artemisiifolia* L.) Pollen after Exposure of the Plants to Elevated Ozone over a Whole Growing Season

**DOI:** 10.1371/journal.pone.0061518

**Published:** 2013-04-18

**Authors:** Ulrike Kanter, Werner Heller, Jörg Durner, J. Barbro Winkler, Marion Engel, Heidrun Behrendt, Andreas Holzinger, Paula Braun, Michael Hauser, Fatima Ferreira, Klaus Mayer, Matthias Pfeifer, Dieter Ernst

**Affiliations:** 1 Institute of Biochemical Plant Pathology, Helmholtz Zentrum München, German Research Center for Environmental Health, Neuherberg, Germany; 2 Research Unit for Environmental Simulation, Helmholtz Zentrum München, German Research Center for Environmental Health, Neuherberg, Germany; 3 Institute of Soil Ecology, Helmholtz Zentrum München, German Research Center for Environmental Health, Neuherberg, Germany; 4 Center of Allergy & Environment München, Technische Universität and Helmholtz Zentrum München, Neuherberg, Germany; 5 Functional Plant Biology, Institute of Botany, Leopold-Franzens-University Innsbruck, Innsbruck, Austria; 6 Department of Natural Sciences and Mechatronics, Hochschule München, University for Applied Science, Munich, Germany; 7 Division of Allergy and Immunology, Department of Molecular Biology, University of Salzburg, Salzburg, Austria; 8 Institute of Bioinformatics and Systems Biology, Helmholtz Zentrum München, German Research Center for Environmental Health, Neuherberg, Germany; University of Delhi South Campus, India

## Abstract

Climate change and air pollution, including ozone is known to affect plants and might also influence the ragweed pollen, known to carry strong allergens. We compared the transcriptome of ragweed pollen produced under ambient and elevated ozone by 454-sequencing. An enzyme-linked immunosorbent assay (ELISA) was carried out for the major ragweed allergen Amb a 1. Pollen surface was examined by scanning electron microscopy and attenuated total reflectance–Fourier transform infrared spectroscopy (ATR-FTIR), and phenolics were analysed by high-performance liquid chromatography. Elevated ozone had no influence on the pollen size, shape, surface structure or amount of phenolics. ATR-FTIR indicated increased pectin-like material in the exine. Transcriptomic analyses showed changes in expressed-sequence tags (ESTs), including allergens. However, ELISA indicated no significantly increased amounts of Amb a 1 under elevated ozone concentrations. The data highlight a direct influence of ozone on the exine components and transcript level of allergens. As the total protein amount of Amb a 1 was not altered, a direct correlation to an increased risk to human health could not be derived. Additional, the 454-sequencing contributes to the identification of stress-related transcripts in mature pollen that could be grouped into distinct gene ontology terms.

## Introduction

Common ragweed (*Ambrosia artemisiifolia* L.) belongs to the family Asteraceae and is native to North America. Ragweed is an important agronomic weed in the USA [1]. However, it causes problems related to human health, as the ragweed pollen is known to carry one of the strongest pollen allergens [2] and causes seasonal allergic rhinitis and asthmatic symptoms in North America [1], [3]. So far, ten different allergenic proteins consisting of different isoforms could be identified in ragweed pollen and were termed Amb a 1 to 10, with Amb a 1 the most important allergen [3]. Their molecular weights range from 9 kDa to 38 kDa, and they are grouped according to their biological differences [3]. Ragweed was introduced from North America to Europe approximately 100 years ago through contaminated seed shipments, which has resulted in the allergic sensitisation of the European population [3]. In Europe, common ragweed is mainly found in the Rhône valley, Hungary, Bulgaria, Northern Italy and Eastern Austria, and it is now spreading in Germany [4] (http://www.Ambrosiainfo.de/53223897640d5c602/index.html).

Climate change will alter plant growth and also influence the onset, period and intensity of pollen production [5]. A relationship between climate, pollen concentration and allergic rhinitis has been reported [6]. Recent warming may be associated with the increased length of the ragweed pollen season in North America [7], and it has been shown that twice the ambient level of CO_2_ results in increased ragweed pollen production [8] and in significant increases in the Amb a 1 allergen [9]. However, there remain lots of open questions about the link between climate change and elevated CO_2_ with public health [5]. Additional, the long-distance transport of pollen must also be taken into account [10].

Increasing air pollution, primarily caused by vehicle traffic, makes pollen allergens more aggressive [11]. The interactions of airborne particles with the surface of pollen or high concentrations of pollutants is leading to a changed pollen morphology [12], and higher concentrations of allergenic proteins were found in the pollen of air-polluted regions [13]. However, there are also contrary reports that have indicated no differences between rural and urban regions or even a greater abundance of an allergenic protein in rural regions [14]. Significant differences in allergenic proteins from birch pollen were also observed in different regions of Europe and in different years [15]. This indicates a complex interaction of different environmental factors on pollen allergenicity that is not triggered by allergenic proteins alone [14], [16].

Tropospheric ozone is one of the most harmful air pollutants and is also relevant to global change [17]. Chronic exposure of vegetation to ground levels of ozone will likely increase during the upcoming decades, and exposure will stay at such levels, increasing risks for vegetation [17]. With regard to pollen, it has been shown that elevated ozone results in decreased pollen germination and pollen tube growth [18]. In recent decades, the effects of ozone have been studied at the physiological, biochemical and molecular biological levels [17]. Numerous transcripts, up-regulated in leaves by ozone, belong to the category “disease and defence”, which also includes pathogenesis-related (PR) protein transcripts [17], [19]. The exposure of rye cultivars and *Lolium perenne* to elevated ozone concentrations during plant growth increased the allergen content in their pollen [20], [21], whereas in different cultivars of *Lolium*, no significant differences in group 5 allergen were evident between control plants and plants grown in ozone concentrations up to 140 ppb [22]. Ozone fumigation of ragweed plants, up to catkin initiation, showed no effects on growth parameters such as leaf area, total biomass or catkin weight. It was concluded that ragweed is insensitive to ozone levels up to 80 ppb [1]. Similar non-significant changes in plant size and pollen amount were found for ragweed grown under controlled conditions, fumigated with 80 ppb (Kanter *et al.*, unpublished). The direct *in vitro* fumigation of ragweed pollen with ozone resulted in a reduced pollen viability, whereas no influence on Amb a 1 protein content and the expression profile of major allergens was observed [23].

Flavonoids, as well as hydroxycinnamic acid-derivatives, are common metabolites of plant pollen, especially the glycosylated types. Flavonoids play a role in fertility, and quercetin has been demonstrated to be an important germination-inducing compound [24]. Additional, these metabolites are known to possess antioxidative and antimicrobial activities. Flavonoids also have UV-B-absorbing properties, thus they protect the pollen, in addition to sporopollenin, during long-range transport [25]. Flavonoids may be involved in the modulation of immune responses, which would also be a key role in the allergenic response to the pollen [26], [27]. The IgE-binding of allergens may be influenced by attached flavonoids [28], and a direct interaction of allergens with biologically important ligands, including flavonoids, has been shown [29], [30]. Air pollution resulted in an increased amount of unknown flavonoid metabolites in the pollen of Cupressaceae, and elevated ozone concentrations resulted in the accumulation of apiin in parsley leaves [31], [32]. Interestingly apigenin, the aglycone of apiin, is able to immunomodulate dendritic cells [27].

Pollen transcriptomic analyses have thus far only been carried out primarily with model plant organisms such as *Arabidopsis thaliana* or *Glycine max* [33], [34]. Furthermore, hierarchical clustering and principal component analysis indicated a clear separation of pollen from other vegetative tissues and clearly defined pollen-specific transcripts [35]. In this study, we describe the results from large scale analyses of ragweed pollen performed by deep pyrosequencing technology. The data include transcripts that were differentially expressed under ambient and twice the ambient level of ozone. An additional focus is the characterisation of allergen-related transcripts and changed allergenic protein abundances upon elevated ozone concentrations. Scanning electron microscopy (SEM) analyses, attenuated total reflectance–Fourier transform infrared (ATR-FTIR) spectroscopy of the pollen wall and phenolic metabolite profiles of ozone-treated ragweed pollen are also presented. The experiment, using realistic outdoor ozone fumigation, allowed the investigation of ragweed over a whole growing season, including the development of pollen and extends studies where only pollen was fumigated with ozone. It provided a link between controlled chamber conditions and analyses of pollen in rural and urban sites.

## Materials and Methods

### Ethics Statement

No specific permits were required for the described studies. Plant material used in this study was grown in exposure chambers under controlled conditions. Initial ragweed seeds were collected in an outdoor stand, for this no specific permissions were required. The sampling location was not privately-owned or protected in any way.

### Plant Growth Conditions

Ragweed seeds were collected from a single plant at an outdoor stand to avoid environmental-dependent epigenetic effects on growth and development [36]. Seeds were applied to standard soil (Floradur®, Bayerische Gärtnerei Genossenschaft, München, Germany) in pots, and plants were cultivated in eight Plexiglass sub-chambers (1.1 m×0.9 m×0.8 m) placed within two phytotron walk-in chambers [37] (Kanter *et al.*, unpublished). (http://www.helmholtz-muenchen.de/en/eus/environmental-simulation-facilities/phytotron/index.html). Thus, four sub-chambers served as technical repetitions of each treatment. During the experiment, the average seasonal course of climatic conditions between May 1^st^ and September 15^th^ were simulated on an hourly basis (Figure S1). The light period was between 14.5 h and 16 h per day (approx. 500 µmol m^−2^ sec^−1^ PPFR with a realistic portion of UV-A during the daily course). The day/night temperatures were 20–30°C/10–20°C, and relative humidity was maintained at 30–50%/80–85% (day/night). Two sub-chambers in each phytotron were fumigated with 40 ppb (control) and 80 ppb ozone for the whole vegetation period, starting on June 19^th^. Watering was carried out by an automated irrigation system. The number of seedlings was reduced to one per pot after three weeks, plants were further grown under normal air for four weeks to acclimate, and ozone treatment was started on June 19^th^. Pollen was continuously collected from July to August 30^th^, using a modified ARACON system (BETATECH, Ghent, Belgium) that covered the male inflorescences. On August 23^rd^ and August 30^th^ the collected pollen samples were stored at −80°C until use.

### Scanning Electron Microscopy (SEM)

SEM was essentially performed as described by Holzinger *et al.* (2009) [38]. Instead of the dehydration procedure, air-dry pollen was gold surface-coated and examined with a Philips XL20 scanning electron microscope (Philips Electronics, Eindhoven, The Netherlands) at 10 kV.

### Attenuated Total Reflectance–Fourier Transform Infrared Spectroscopy (ATR-FTIR)

ATR-FTIR spectra of *Ambrosia* pollen fumigated with ambient and twice ambient ozone were recorded using a Bruker Tensor 27 spectrometer equipped with the ATR accessory ZnSe crystal cell attached to the spectrometer with a liquid nitrogen cooled mercury cadmium telluride detector and a KBr beam splitter. ATR–FTIR spectra (3,050–900 cm^−1^) were taken with 4 cm^−1^ resolution and a sampling time of 32 scans [39]. Frozen pollen samples were placed onto the crystal cell and gently compressed during measurement.

### Analyses of Phenolic Metabolites

Frozen pollen was extracted with phosphate buffered saline (PBS), and the residue was then extracted with methanol. Reverse-phase high-performance liquid chromatography (RP-HPLC) separation of the PBS-soluble and methanol-extractable phenolics was performed as described previously [40].

### Enzyme-linked Immunosorbent Assay (ELISA)

*Ambrosia* pollen extracts were prepared from 100 mg pollen by shaking in 1 ml 1× PBS buffer, pH 7.4 for 1.5 h at room temperature followed by centrifugation at 10,000 *g* for 15 min. The total protein concentration of the extracts was determined in triplicate by a Bradford assay. For direct ELISA, maxisorp plates (NUNC, Roskilde, Denmark) were coated with 50 µg ml^−1^ *Ambrosia* pollen extract in 1× PBS buffer, 50 µl per well and incubated overnight at 4°C. Plates were blocked with Tris buffered saline (TBS), pH 7.4, 0.05% (v/v) Tween and 0.5% (w/v) BSA for 2 h at room temperature and incubated with a murine monoclonal IgG1 anti-natural Amb a 1 antibody, diluted 1∶1,000, for 1.5 h at 37°C. After washing with TBS, pH 7.4 and 0.05% (v/v) Tween, plates were incubated with an alkaline phosphatase-conjugated rabbit anti mouse IgG+IgM antibody (Jackson Immuno Research, PA, USA), diluted 1∶1,000, for 1 h at 37°C and 1 h at 4°C. 4-Nitrophenyl phosphate (Sigma-Aldrich, MO, USA) at 10 mM was used as substrate, and the OD was measured at 405/492 nm. All measurements were performed in triplicate. The results are presented as the mean OD values.

### RNA Isolation

Total RNA was isolated from 50 mg pollen using a modified Qiagen RNeasy Mini Kit protocol. In brief, pollen, together with 150 µl of RLT buffer, was transferred to 2 ml tubes containing 1.4 mm ceramic spheres, 0.1 mm silica spheres, and a single 4 mm glass sphere. The pollen was homogenised eight times at 6.5 ms^−1^ for one minute each on dry ice in a FastPrep 24 machine (MP Biomedicals, Eschwege, Germany). Then, another 600 µl RTL buffer was added, and the tube was shaken again. One volume of chloroform was added and incubated for 10 min on a shaker. After centrifugation, the supernatant was transferred to a new reaction tube and mixed with 0.5 volumes of ethanol by gently inverting. The solution was transferred to RNeasy columns (RNeasy Mini Kit, Qiagen, Hilden, Germany) and centrifuged for 15 s at 10,000 *g*. The column was incubated with 450 µl RW1 buffer for 5 min and then centrifuged. The flow-through was discarded, and DNase digestion was performed following the manufacturer’s instructions (RNase-Free DNase Set; Qiagen, Hilden, Germany). Subsequently, the column was incubated twice with 500 µl RPE buffer for 2–3 min each. Drying and elution of the RNA was performed according to the user manual (RNeasy Mini Kit, Qiagen).

### Titanium Sequencing

Total RNA was extracted from three pooled pollen samples (50 mg each, grown under 40 ppb and 80 ppb ozone), quantified and analysed using a NanoDrop ND1000 at wavelengths of 230, 260 and 280 nm. Two non-normalised cDNAs, ready for GS FLX Titanium sequencing were prepared by Vertis Biotechnology AG (Freising, Germany). 454-sequencing of both cDNAs was carried out using the Titanium Genome Sequence Systems according to the manufacturer’s instruction (Roche Diagnostics GmbH, Mannheim, Germany).

### Bioinformatic Analysis

The analysis of the original 454-read sets are given in Table S1. To avoid short fragments during the assembly process, the fraction of small 454-reads (24%) was removed and excluded from the assembly process. For the assemblies, the Newbler v.2.5.3 transcriptome assembly (-cDNA option) and default parameters were used. In order to quantify expression levels of *Ambrosia* transcripts in the ozone and control treated plants, the available 454-reads of the individual samples were aligned to the assembled isotigs by using vmatch v2.1.7 (-l 40 -e 1 -identity 98) (http://www.vmatch.de/). Returned alignments were filtered and for each read only alignments with maximum e-value were considered for further analysis (workflow: Figure S2). Transcript expression levels were quantified in reads per kilobase of exon model per million mapped reads (RPKM) that measures the read density normalized for RNA length and the total number of reads in the experiment [41]. Thereby, individual reads mapped to multiple isoforms were uniformly divided to all mapping positions. The matched *Ambrosia* transcripts were further analysed in order to detect genes that were either matched by sequences of both groups or exclusively matched one group.

For each of the Newbler isogroup with two or more alternative splicing variants, the isotig with longest predicted protein sequence was selected as gene representative. Then, putative orthologous gene pairs between assembled *Ambrosia* genes and *Arabidopsis* (TAIR10) were identified based on a two level homology search against the *Arabidopsis* TAIR10 gene set (BLASTP, e-value ≤1e−5) (workflow: Figure S2):

- bi-directional BLAST searches (*Ambrosia vs. Arabidopsis* and *Arabidopsis vs. Ambrosia*, respectively) were performed.
- *Arabidopsis* genes, which were exclusively matched by the first best BLAST hit of one distinct *Ambrosia* isotig.

Moreover, sequence similarity searches against the NCBI non-redundant protein database (July 2011, BLASTP) excluding all *Arabidopsis* genes, a set of known *Ambrosia* allergens (BLASTN) and a set of known plant allergens (NCBI search with keywords “viridiplantae” and “allergen”) (BLASTP) was performed. Only alignments with e-value ≤1e−5 were considered as significant and the first-best hit (fbh) against the reference data sets extracted for each *Ambrosia* sequence. In total, 1,542 of 2,877 (54%) *Ambrosia* representative transcripts showed conserved sequence homology to at least one reference data set (Table 1).

**Table 1:** Comparison of 454 transcriptome assembly against reference protein databases.

	*Arabidopsis* (TAIR10)	*Ambrosia* Allergens	NCBI Plant Allergens	NCBI non. red.	Total (non. red.)
**Reference proteins**	35,386	47	7,384	399,458	–
**Matched reference proteins (any hit)**	7,831	30	587	22,949	
**Matched reference proteins (fbh)**	1,008	12	62	1,260	–
**Mapped ** ***Ambrosia*** ** transcript representatives**	1,415	21	133	1,532	1,542

Raw sequencing data and assembly data were submitted to the European Nucleotide Archive (ENA) with the project accession: PRJEB1470.

## Results

### Scanning Electron Microscopy (SEM)

**Figure 1:**
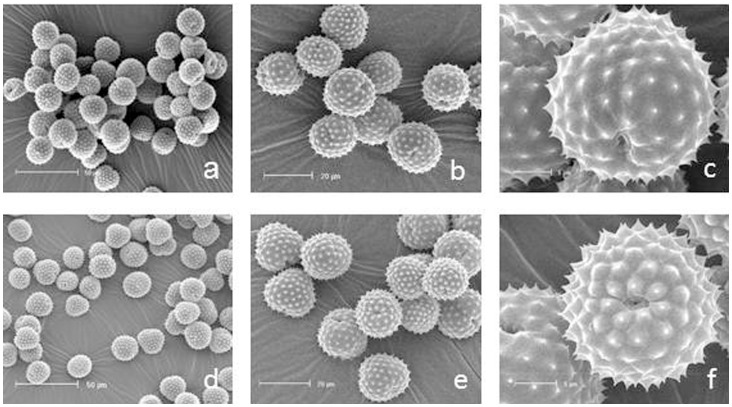
Scanning electron microscopy of ragweed pollen exposed to different ozone concentrations: (a–c) 40 ppb ozone, (d–f) 80 ppb ozone. Bars: a, d 50 µm; b, e 20 µm; c, f 5 µm.

SEM showed the expected shape of ragweed pollen grain (Figure 1). The mean size of the roundish pollen was in the range of 15–24 µm and exhibited the typical circular apertures. SEM analyses revealed that the exine of the pollen was coated with small cone-shaped needles. According to SEM photographs the pollen shape appeared to be similar and no significant differences in pollen size between the control and ozone-exposed plants could be detected (ozone: 20.57±1.64 µm, n = 100; control: 21.15±1.45 µm, n = 100).

### ATR-FTIR Analysis of Ragweed Pollen

**Figure 2:**
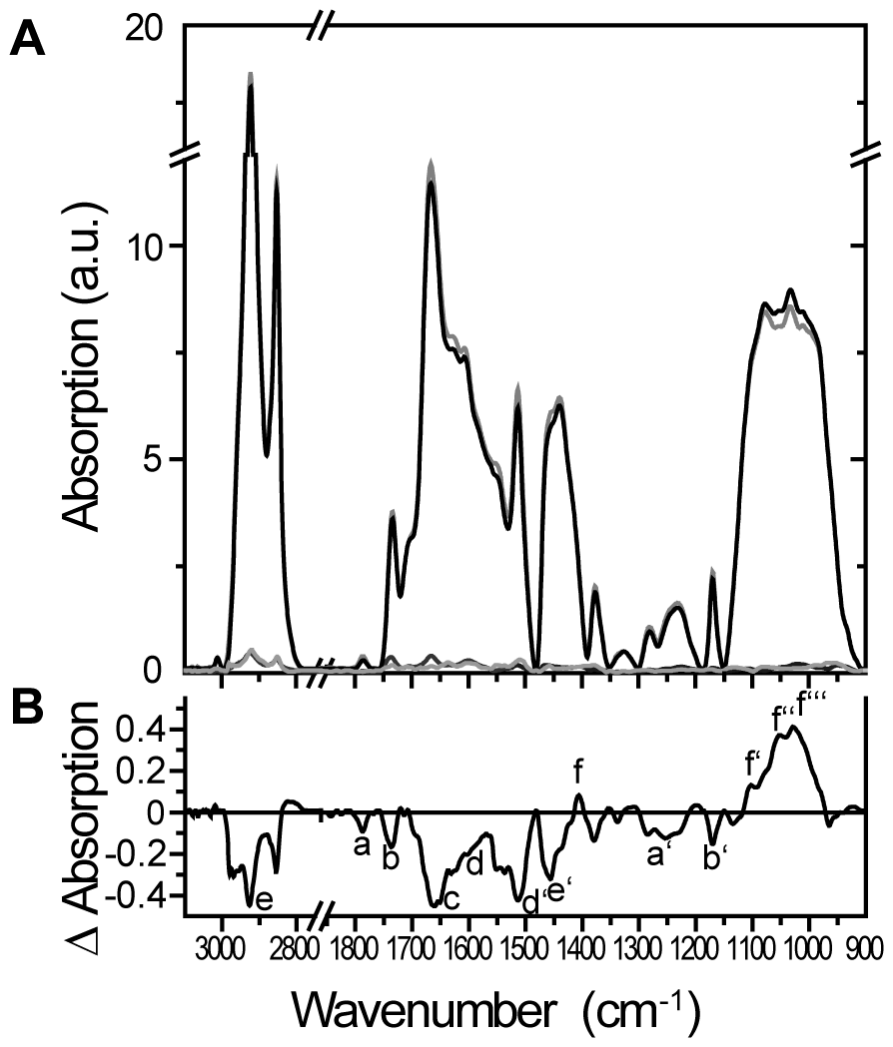
ATR-FTIR spectra of ozone- and control-treated *Ambrosia artemisiifolia* pollen. **A** shows the averaged absorption spectra of ozone-treated (black; n = 5) and control pollen (grey; n = 6) in a range of wavenumbers between 900–3050 cm^−1^. Standard errors for these spectra are stated at the bottom of part A in black and grey, respectively. **B** indicates the Δ-absorption of ozone spectra minus control spectra. Small letters stated in part B are further described in Table 2.

The ATR-FTIR spectra of *Ambrosia* pollen treated with elevated ozone compared to the control are shown in Figure 2 and further described in Table 2. The two strong absorption bands located at ∼2850 and ∼2920 cm^−1^ originate from the asymmetric and symmetric stretching vibrations of the methyl and methylene groups present in hydrocarbon chains, for example, in glycerolipids and wax hydrocarbons (Figure 2B; e). The minor IR absorption band recorded at ∼1470 cm^−1^ may be assigned to methylene deformation vibrations; its intensity is usually much weaker compared to the CH_2_-stretching vibrations (Figure 2B; e’). As shown by the IR difference spectra (Figure 2B), the bands at ∼2920, ∼2850 and ∼1470 were significantly decreased in pollen samples that were treated with elevated ozone, indicating reduced numbers of methylene groups, e.g., in lipid and wax components. Absorptions recorded between 1660–1500 cm^−1^ corresponded to the so-called aromatic domain of the samples, such as in phenolic compounds, for example, in the aromatic rings of sporopollenin components (Figure 2B; d, d’). In addition, the proteins’ amid I absorption was recorded between 1680–1620 cm^−1^, the exact vibrational frequency of which depended on the CO and NH conformational states (Figure 2B; c). The strong difference in absorption between ozone-treated and control pollen, with a peak intensity at ∼1512 cm^−1^ and a shoulder at ∼1603 cm^−1^, suggests a relative decrease of phenolic compounds of sporopollenin in ozone-treated pollen [42]. Finally, a set of different absorption bands were recorded in the 1200–950 cm^−1^ region (Figure 2B; f’–f’’’). The shape and relative intensities of the different shoulders located at approximately 1100, 1048 and 1025 cm^−1^ can be clearly assigned to C-O and O-Η deformations vibrations in secondary alcohols and define the polysaccharide absorption range. Specifically, the IR frequencies of ring and side group vibrations of pectin (∼1100, ∼1047, ∼1017 cm^−1^) have been assigned by model studies to rhamnogalacturonic acid at ∼1043 cm^−1^, arabinan at ∼1100 cm^−1^, glucan at ∼1026 cm^−1^ and glucomannan at ∼1034 cm^−1^ [43]. The absorption intensities of the pectin vibrations have apparently increased when compared to the lipid and protein absorptions, as is obvious from both the IR absorption and difference spectra (Figure 2B, f’–f’’’).

**Table 2:** ATR-FTIR analysis – explanation of labels from Figure 2.

Label in Figure 2	Band origin	Wavenumber of absorption [cm^−1^]	Change due to ozone-treatment
	**Pectin:**		
a	COO-R, R = Me, = Ac	1750–40, 1770–60	−
a’	COC	1280, 1250	−
	**Lipid:**		
b	Acylester: COOR	1740–20	−
b’	COC	1170	−
	**Protein: amid I:**		
c	CO, NH	1680–20	−
	**Sporopollenin:**		
d, d’	aromatic, phenolic stretches: CC, CO	1603, 1512	
	**Glycerolipid, wax:**		
e, e’	methylene: CH_2_	2920, 2850, 1465	− −
	**Pectin:**		
f	carboxyl: COO^–^	1410	+
f’, f’’, f’’’	pyranoid ring: CC, COH, CH	1100, 1048, 1025	+ +

ATR-FTIR analysis of ozone-treated pollen of *Ambrosia artemisiifolia* compared to the control. Labels stated in the figure are shown, as well as the band origin and wavenumbers of absorption. Changes in absorption between the two treatments are stated. A reduction in the ozone sample compared to the control is indicated by a minus sign (− or − −), whereas a+or++ reflects an increase in the ozone-treated pollen.

The absorption bands at ∼1770 and ∼1740 cm^−1^ may originate from the C = O stretching vibration of the carbonyl group of the pectin’s acetylester bond (Figure 2B; a) and either of the carbonyl of the pectin’s methylester or the wax and lipid’s acylester (Figure 2B; b), respectively [44]. Acetylated pectates also have a strong IR band at ∼1250 cm^−1^ (Figure 2B; a’), which is assigned to the (υCOC) of acetyl, whereas methylated PecAc has been proposed to have three IR bands at ∼1280, ∼1250 and ∼1220 cm^−1^ (Figure 2A) [45]. Interestingly, the pectin’s acetylester absorption at ∼1770 cm^−1^ is decreased in ozone-treated pollen when compared to the pectin’s pyranoid ring group absorptions (at ∼1100, ∼1047, ∼1017 cm^−1^), perhaps indicating the deesterification of pectate [45]. It cannot be determined whether de-methylation of pectin occurred due to the overlapping of different IR active groups in the range of the pectin’s methylester. Nevertheless, elevated ozone resulted in the apparent decrease of the lipid wax, protein and sporopollenin components as compared to the pectin layer. It should be stated, however, that additional analytics are needed to unequivocally and quantitatively determine the changes in pollen surface constituents upon exposure to elevated ozone. To test whether an increase of pectin compared to the lipid wax could be true in pollen, mixtures of different lipid/pectinC weight ratios have been analysed by ATR-FTIR (Figure S3). The doubling of pectin C relative to the lipids resulted in a loss of absorption at ∼2924 and ∼2854 cm^−1^ (major absorption bands of the lipid acyl chain group) and an increase of absorption with maximum at ∼1022 cm^−1^ (major absorption band of secondary alcohols of pectin) as shown in the difference spectrum (Figure S3, b). The Δ-IR-absorption obtained by merely raising the pectinC content in the lipid/pectin mixture is similar to the Δ-IR-absorption of ozone- and control-treated *Ambrosia* pollen, namely an increase in the polysaccharide absorption range. Naturally, in the complex mixture the resolution of the other medium and minor pectin absorption bands is not achieved. Thus, the similarity of the difference spectra of the two component mixture as compared to the complex mixture of residual components in pollen walls further supports the notion of an increase in polysaccharide, particularly pectin.

### Secondary Metabolites

**Figure 3:**
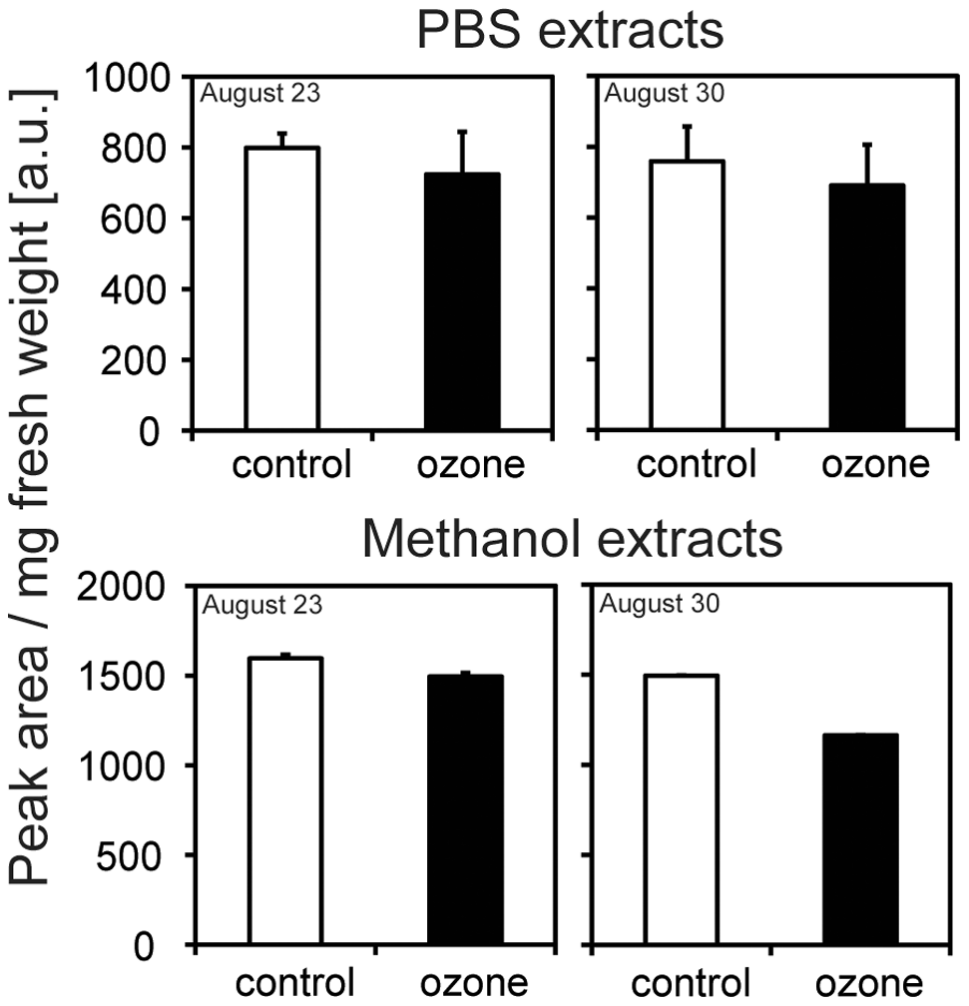
Accumulation of total phenolics analysed. Plants were fumigated with 40 ppb (control) or 80 ppb ozone; bars indicate ± SD; n = 7.

In PBS-soluble extracts, 17 prominent compounds were evaluated. The highest amounts were found for quercetin derivatives. Methanol-extractable phenolics showed additional 12 prominent compounds, characterised as hydroxycinnamic amides according to their typical diode-array spectra. On the basis of peak areas obtained at 280 nm, total amounts of individual compounds for the two harvest time points together with typical RP-HPLC diagrams are given in Figure S4. No significant changes were observed between control- and ozone-treated samples. Summarising the amount of PBS-soluble as well as methanol-extractable metabolites, a slight, but not significant ozone-dependent reduction, as well as a reduction in several metabolites was observed over time (Figure 3, Figure S4).

### Roche Titanium Sequencing and Assembly of the 454-Reads

Two sequencing runs (ozone and control) yielded a total of 1,242,132 raw reads. The length frequency distribution ranged from 40–772 bp (Figure S5a). To avoid short fragments during the assembly process, the fraction of small 454-reads (24%) were removed and excluded from the assembly process (Table S1). A total of 982,467 reads, equivalent to a total sequence length of approximately 324 Mb, was then used for the assembly process. Using the cleaned sets of 454-reads, an ensemble transcriptome assembly was performed using Newbler v2.5.3. This resulted in 5,720 contigs and 49,729 singletons. Singletons were not used for further analysis. Furthermore, the Newbler Transcriptome Assembler combined the assembled contigs into 5,052 individual transcripts (“isotigs”) that were collected in 2,938 “isogroups” based on shared branches in the underlying contig graph (Table S2). Open reading frames (ORFs) were predicted for 4,950 isotigs (encoded by 100 or more nucleotides). The longest ORF was chosen as most probable protein sequence of an isotig and used for further analysis. Isotigs without protein sequence were discarded.

In order to quantify expression levels of *Ambrosia* transcripts in the ozone and control treated plants, the available 454-reads of the individual samples were aligned to the assembled isotigs by using vmatch v2.1.7 (−l 40 -e 1 -identity 98) (http://www.vmatch.de/). Returned alignments were filtered and for each read only alignments with maximum e-value were considered for further analysis. As summarised in Table S3 and visualised in Figure S2, a total of 299,051 454-reads (52%) out of 576,199 raw reads of the ozone-treated sample and 349,759 454-reads (53%) out of 665,933 raw reads of the control sample were matched against the 5,052 ensemble isotigs and aligned to 4,923 and 4,975 *Ambrosia* transcripts, respectively. Thereby, the majority (81% and 82%) of the matched 454-reads were unambiguously mapped to a unique isotig. However, almost all matches of multiple mapped 454-reads (98%) were observed for isotigs of the same isogroup.

**Figure 4:**
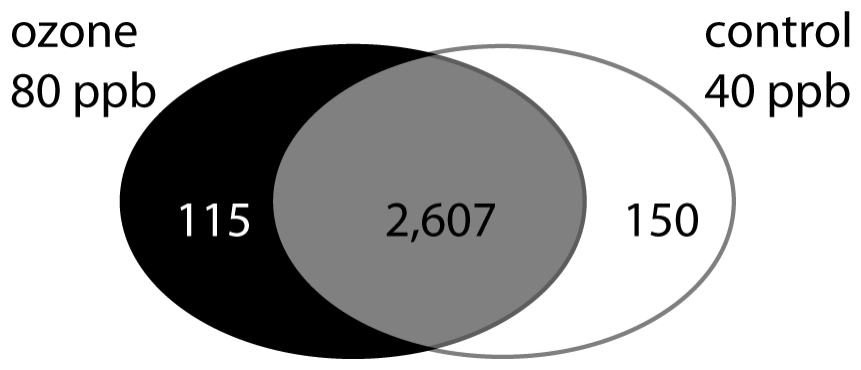
Venn diagram. Common and differently matched *Arabidopsis* genes. Number of *Arabidopsis* genes that matched by either sequences of both groups or sequences of one group exclusively.

In order to filter low-abundance transcripts different RPKM thresholds between 1 RPKM and 10 RPKM were applied (Table S4). More than 90% (2,607) of the assembled *Ambrosia* transcripts are commonly expressed with at least 7 RPKM (lower 5^th^ percentile of transcript abundances (Table S5), in the ozone and control treatment (Figure 4) as well as 116 ozone specific genes and 150 control-specific genes, respectively, could be identified. To test the reliability of RPKM-values several transcripts with high and low RPKM-values were further analysed by quantitative RT-PCR. Even so a plant to plant variability was detectable a positive correlation of qRT-PCR results to RPKM-values could be detected with r^ = ^0.984 (Pearson-correlation; Figure S6).

### Comparison of the 454-Transcriptome Assemblies to the *Arabidopsis* Gene Set

For each of the Newbler isogroup with two or more alternative splicing variants, the isotig with longest predicted protein sequence was selected as gene representative. Then, a total of 957 putative orthologous gene pairs between assembled *Ambrosia* genes and *Arabidopsis* (TAIR10) were identified based on a two level homology search against the *Arabidopsis* TAIR10 gene set (BLASTP, e-value ≤1e−5) (workflow: Figure S2):

- bi-directional BLAST searches (*Ambrosia vs. Arabidopsis* and *Arabidopsis vs. Ambrosia*, respectively) were performed and 774 best bidirectional hits (bbh) identified.
- The remaining hits 233 matched *Arabidopsis* genes were further analysed and 183 reference genes were detected, which were exclusively matched by the first best BLAST hit of one distinct *Ambrosia* isotig.

**Figure 5:**
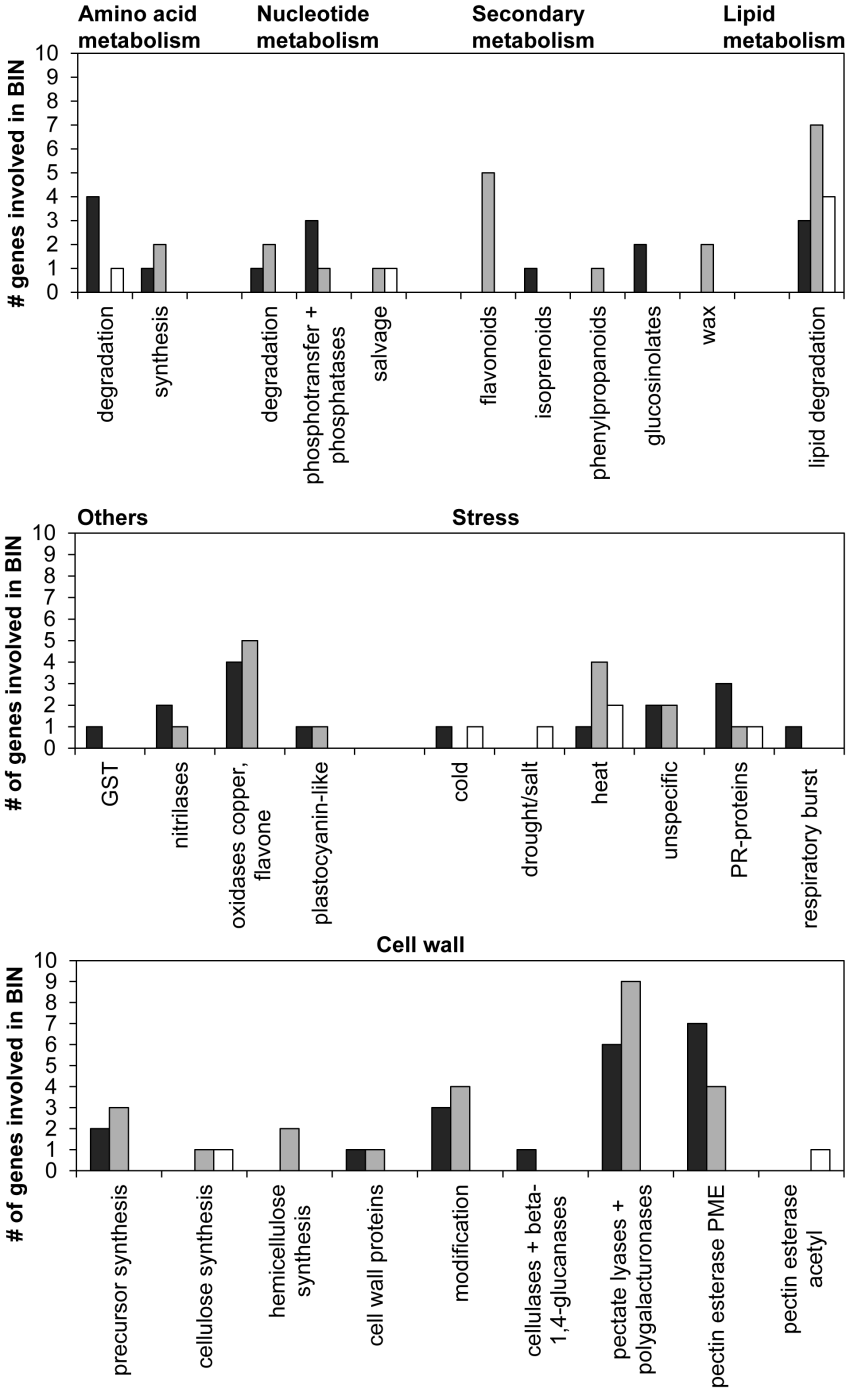
Interesting BIN terms detected by MapMan [**46**]. *Arabidopsis* sequence matches were grouped due to their log_2_-fold-change value to three groups: black = induction by ozone (log_2_>1), grey = common (log_2_≤ −1; log_2_≥1), white = repression by ozone (log_2_< −1). For these groups, the number of transcripts according to the BIN term is stated. Only matches with e-value ≤1 e−5 and RPKM ≥7 were taken into account.

A detailed table, including all tagged *Arabidopsis* genes, the gene description, alignment details, mapped *Ambrosia* reads and RPKM-values, is provided as a supplementary Excel file (Table S6). For quantification RPKM-values were used and a log_2_-fold-change of treatment against control was calculated. For genes with no expression in one group, the RPKM-value was set to 0.1 to calculate the fold-change. To obtain stringent values and to avoid “false positive” interpretation of log_2_-fold-changes, all transcripts with a RPKM-value less than 7 were considered to be not expressed. Transcripts were then analysed via MapMan [46] to group them to several functional categories (BIN-codes). Analysing several BINs involved in plant stress showed a couple of genes induced or repressed by ozone (Figure 5 and Table S7). Transcripts with homology to genes involved in respiratory burst, including a oxidoreductase acting on NAD(P)H, a monodehydroascorbate reductase from the term “redox” and a gene for a glutathione-S-transferase, were up-regulated in ozone-treated pollen up to a log_2_-fold change of 2.23, whereas two glutaredoxins found within the BIN term “glutathione” were only slightly induced by ozone (max. log_2_-fold change: 0.67), which are involved in coping with the elimination of reactive oxygen species. In addition to transcripts with homology to genes involved in the redox state of the cell, also transcripts with oxidoreductase activity from the term “oxidases copper, flavone” (max. log_2_-fold change: 3.16) showed higher transcript levels in the ozone-treated pollen. For the BIN term “secondary metabolites” a slight repression of transcripts involved in wax biosynthesis, such as the membrane bound O-acyl transferases (max. log_2_-fold change: −0.99) and an increase of transcripts involved in “glucosinolates” and were recognised due to elevated ozone (max. log_2_-fold change: 2.81).

For the BIN term “cell wall”, the pectin methyl esterases were clearly induced due to the ozone treatment (max. log_2_-fold change: 3.39) and the expression of the pectate lyases was also elevated under ozone treatment (max. log_2_-fold change: 1.42). Beside this also transcripts involved in “precursor synthesis” and “modification” were increased due to the ozone fumigation. Another interesting group influenced by ozone treatment was the BIN term “misc.nitrilase”, which included several berberine bridge enzyme-like proteins (max. log_2_-fold change: 2.33). This enzyme family is known to show allergen action, e.g., in Timothy grass (Phl p 4) or celery (Api g 5) [47], and might also be a candidate for a possible allergen.

### Comparison of the 454-Transcriptome Assemblies to the NCBI Non-Redundant Protein Database

**Figure 6:**
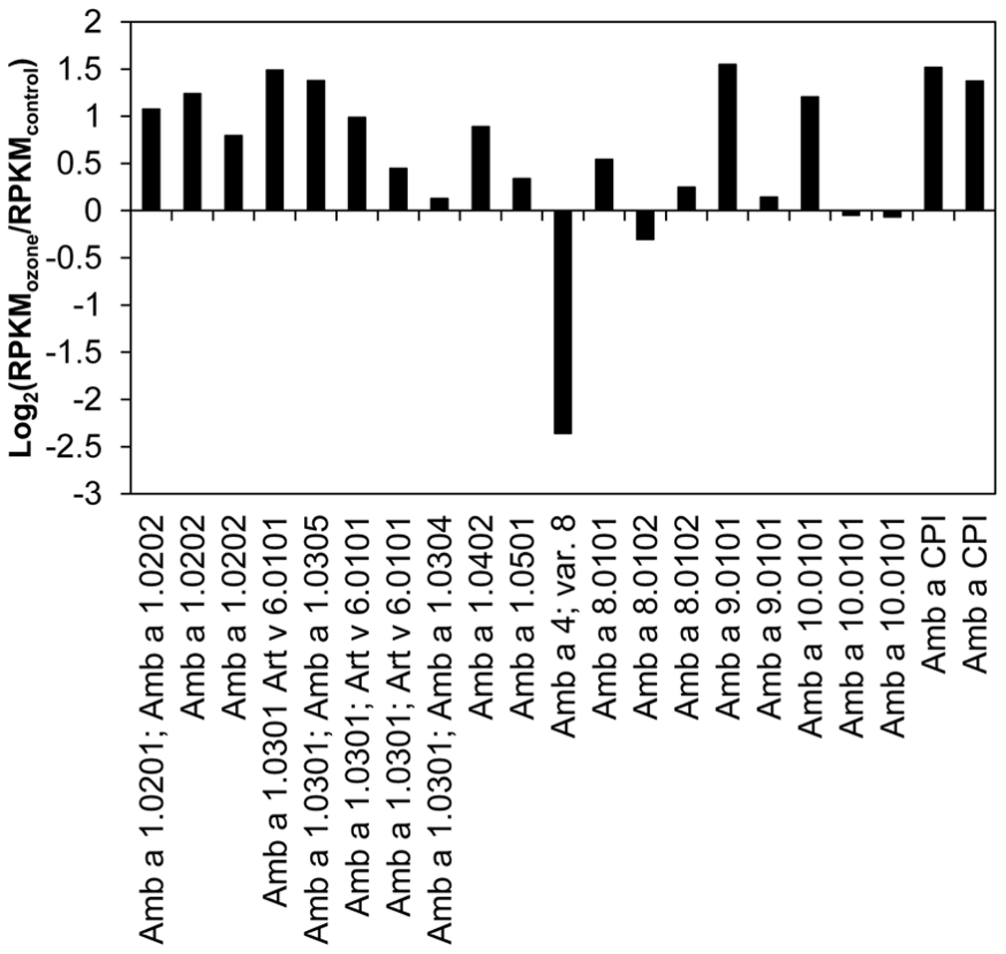
Log_2_-fold changes (RPKM_ozone_/RPKM_control_) of known *Ambrosia artemisiifolia* allergens. All data presented are isotig specific and normalized on RPKM.

A comparison of the *Ambrosia* transcriptome to the *Arabidopsis* gene set will not reveal possible allergenic proteins. A BLAST comparison of all *Ambrosia* sequences against the NCBI non-redundant protein database, against a set of known *Ambrosia* allergens and against a set of known plant allergens (NCBI; keywords “viridiplantae” and “allergen”) was additionally performed, and only first best hits with e-value ≤1e−5 were considered to be significant. Considering both groups, 23,566 matches were found, taking only first best hits into account 1,322 fbh-matches were found. These matches were mapped to 1,517 *Ambrosia* transcripts from group 1 and 1,521 from group 2. Within these pre-filtered datasets, only allergenic hits were further analysed. This resulted in 152 hits for group 1 and 151 hits for group 2 (Table 3, Table S8). The primary homologies were found to the allergen families of polcalcin (calcium-binding protein, EF hand domain), polygalacturonase, pectin methylesterase and pectate lyases. Regarding allergens from *Ambrosia*, several isoforms of Amb a 1 (pectate lyase), Amb a 8 (profilin) and Amb a 9 (calcium-binding protein), Amb a 10 (calcium-binding protein), one Amb a 4 (defensin-like) and Amb a CPI (cystatin) were found. Changes in the transcript amounts upon ozone treatment are given in Figure 6, represented by log_2_(RPKM_ozone_/RPKM_control_)-values for each allergen annotated isotig. The expression of most *Ambrosia* allergen ESTs was higher in ozone-treated pollen, up to 2.9-fold greater than the control, whereat an isotig- or splice form- specific expression was detectable. Of these *Ambrosia* allergens, the major allergen Amb a 1 and isotigs for the allergen Amb a CPI were expressed at the highest level represented by the high RPKM-values.

**Table 3:** Allergens detected by 454-sequencing in *Ambrosia artemisiifolia* pollen.

Protein function	Allergen *Ambrosia*	Homology to other plant allergens found in
**Actinidin**		*Actinidia deliciosa*
**Aldoketomutase**		*Oryza sativa* var. *japonica*
**Bet v 1 like**		*Vitis vinifera; Selaginella moellendorffii*
**Bromelain**		*Ananas comosus*
**Calcium-binding**	Amb a 9; Amb a 10	*Arachis hypogaea; Betula pendula; Glycine max; Juniperus oxycedrus; Medicago truncatula; Oryza sativa* var. *japonica; Vitis vinifera*
**Cystatin**	Amb a CPI	*Actinidia deliciosa*
**Defensin-like**	Amb a 4	
**Esterase**		*Hevea brasiliensis; Oryza sativa* var. *japonica*
**Expansin**		*Solanum lycopersicum*
**FAD-linked oxidoreductase BG60**		*Cynodon dactylon*
**L-ascorbate oxidase-like protein grass** **pollen group 4 allergen**		*Cynodon dactylon*
**LTP**		*Platanus orientalis*
**Ole e 1-like**		*Arabidopsis thaliana; Ricinus communis*
**Papain**		*Carica papaya*
**Pectate lyase**	Amb a 1	*Arabidopsis thaliana; Artemisia vulgaris*
**Pectin acetylesterase**		*Arabidopsis thaliana*
**Pectin methylesterase**		*Elaeis guineensis; Salsola kali*
**Polygalacturonase**		*Elaeis guineensis; Olatanus x acerifolia*
**PR-1**		*Artemisia vulgaris; Oryza sativa* var. *japonica*
**Profilin**	Amb a 8, Amb a D03	
**Protein kinase**		*Salsola kali*
**LEA III-like pollen allergen**		*Corylus avellana*
**Small rubber particle protein**		*Hevea brasiliensis*
**Thioredoxin; peroxiredoxin**		*Arabidopsis thaliana; Brassica rapa*
**Vilin**		*Nicotiana tabacum*
**Xyloglucan endotransglycosylase**		*Cryptomeria japonica*

Table 3 shows the protein function and common names of *Ambrosia* allergens detected by 454-sequencing in ragweed pollen. Also plants with homologies to other known allergens are indicated. Only first best hits with e-value ≤1e−5 were considered as significant. For further detail see supplementary Table S8.

### ELISA

**Figure 7:**
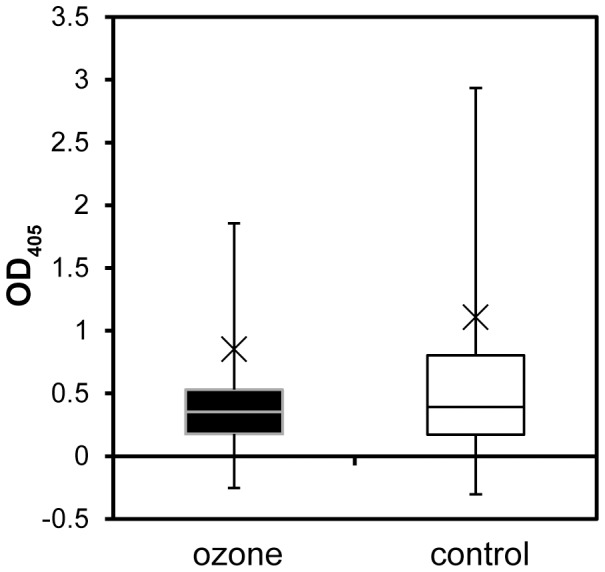
Box-Plot of a direct ELISA for the major allergen Amb a 1 of *Ambrosia* pollen extracts. 50 µg ml^−1^ total protein were coated. Cross indicates the mean value; ozone n = 14, control n = 19.

Pollen protein extracts from ragweed plants grown either under control conditions or elevated ozone were analysed for their Amb a 1 content via direct ELISA. First, the antibody sensitivity in detection of Amb a 1 was tested by immunoblot. *Ambrosia* pollen extracts from 7 different groups were analysed via immunoblot using polyclonal rabbit anti Amb a 1 sera, as well as murine monoclonal mAB anti Amb a 1 A39 (Figure S7). As the murine monoclonal antibody anti Amb a 1 A39 is more sensitive in recognition to Amb a 1 and its cleavage products alpha-chain and beta-chain, A39 was used for analyses of all *Ambrosia* pollen extracts via direct ELISA. As it is seen in Figure 7, no significant differences could be detected between the different treatments, indicating that twice the ambient ozone might not have an influence on the production of the major ragweed pollen allergen Amb a 1.

## Discussion

Once ozone enters the plant cell, ozone rapidly reacts with cell wall components and lipids to form reactive oxygen species (ROS), including singlet oxygen, hydrogen peroxides and hydroxyl radicals [48]. This leads to oxidative stress and changes in the redox potential, with which the pollen has to cope. Genes encoding proteins associated with plant stress response were elevated, including enzymes involved in detoxifying ROS and pathogen-related proteins, similar to as it was seen in beech [19]. ESTs for a monodehydroascorbate reductase (MDAR) as well as gluthatione-S-transferase and a thioredoxin were activated due to elevated ozone. He *et al.* (2006) reported an increased level of MDAR after 10 days of elevated ozone in leaves of *Ginkgo biloba*, followed by a decrease of MDAR after 40 days of treatment [49]. Dehydroascorbate reductase activity was lower than the control after ten days, followed by an increase and then by a decrease after 70 days of treatment, leading to the conclusion that the protection system itself became affected after prolonged treatment and resulted in lowered protection [49], also in this study some of the transcripts involved in the Bin-term “Redox” were found to be repressed.

With regards to secondary metabolites, ESTs involved in flavonoid biosynthesis were found to be slightly repressed. This observations for flavonoids was concordant with results found in beech saplings [19]. Furthermore, an EST involved in isoprenoid biosynthesis, of the mevalonate pathway, was repressed due to ozone treatment. This is interesting because isoprenoids such as isoprene and monoterpenes are known to protect leaves from oxidative damage [50]. Moreover almost no changes were found for ESTs involved in the biosynthesis of polyamins, such as putrescine and spermidine, which are known to play various roles in response to plant stress [51]. Only one arginine decarboxylase (ADC) homologous EST, the key enzyme in plant stress polyamine synthesis, was elevated [52]. A strong positive correlation between ozone exposure and ADC induction was reported for barley and tobacco [53], [54]. Additionally, ESTs involved in wax biosynthesis were reduced in pollen fumigated with elevated ozone. This might be concordant to structural degradations of epicuticular wax observed on the needles of Norway spruce [55]. Ozone stress showed a clear influence on the cell structure, such as the components of the cell wall. An EST for cellulose synthase was repressed, whereas a glycosyl hydrolase found in the BIN “cell wall.degradation.cellulase” was strongly induced due to ozone fumigation. Similar to results in the literature, some ESTs for the cell wall-modifying enzyme xyloglucan endotransglycosylase-hydrolase (XTH), playing a role in plant development, were elevated [19], [56]. With regard to XTH, it has been shown that it reacts to a broad range of abiotic stresses, and it is influenced by the stress hormone ethylene, suggesting a role in the early events of the stress-related defensive response [56]. With ozone treatment, several ESTs coding for enzymes involved in the biosynthesis of cell wall precursors of the hemicelluloses and pectins were induced. These might be involved in cell wall repair or in cell wall stiffening to provide better protection for the pollen. It has been shown for several plants, such as poplar, birch and alder, that cell wall thickening and increased pectin levels of mesophyll cells occur under ozone treatment [57]. A large number of transcripts for pectin modifying enzymes, such as pectate lyases and pectin methylesterases, were increased in the ozone-treated pollen. Pectin might also play a role in pollen protection because pectin methylesterases (PMEs) are involved in cell wall extension and stiffening [58], and de-esterification of pectin has been previously described as taking place during pollen development and supposedly leads to a more rigid form of pectin [59]. It is known that the PME de-esterified homogalacturonan can bind class III peroxidases that might initiate lignin polymerisation [60] and that PMEs react to several biotic and abiotic stresses summarised by Pelloux *et al.* (2007) [61]. Because the carbon source is limited in pollen, it appears to be that the only defence or antioxidative mechanisms that are activated are not cost or energy intensive. Therefore, it is possible that the reconstruction or stiffening of the cell wall requires less in energy and carbon sources than the production of flavonoids, isoprenoids or waxes. Moreover, differences in the rigidity of the cell wall were not detectable by our SEM investigations and only a detailed chemical analysis could elucidate this question.

ATR-FTIR analysis of elevated ozone- and control-fumigated pollen provided insight for the components of the pollen surface. We detected a clear reduction in the FTIR bands of ozone-treated pollen corresponding to glycerolipids or waxes, which is in line with results obtained from the transcriptomic analysis. The reduced intensities of the FTIR peaks corresponding to the acetylester of pectin were detected, in contrast to the increased absorption of the pyranoid ring group of pectin, indicating that de-esterification may have occurred due to ozone treatment. From the FTIR data, it is not possible to determine whether additional de-methylation of pectin took place because lipids are clearly reduced due to ozone treatment, and the carbonyl of the pectin’s methylester and the lipid’s acylester both contribute the absorption band at ∼1740–20 cm^−1^ (Figure 2). However, as the transcriptomic data also indicate de-methylation, it is possible that this peak corresponds more to pectin than to the lipids. ESTs for wax biosynthesis are reduced in the ozone-fumigated pollen, and a reduction in wax compounds is also seen by FTIR analysis. This is concordant with studies on spruce needles that showed a structural degradation of epicuticular wax and a reduced wax layer around the stomata cells due to increased ozone fumigation [55].

Although the transcription level of some isotigs of the major allergen Amb a 1 were elevated in the pollen from ozone-treated (80 ppb) plants, there was no significant difference seen in the protein level, as tested by direct ELISA. This was also observed by Pasqualini *et al.* (2011), who analysed mature pollen after seven days of ozone fumigation, indicating that Amb a 1 itself is not influenced by elevated ozone [23]. Prior studies on the allergen content of plants grown under the influence of gaseous pollutants showed heterogeneous results. In polluted areas *Zinnia* pollen showed an increased allergenicity and in *Cupressus arizonica* pollen a higher expression of Cup a 3, a PR-5 protein, was found [13], [62]. In studies on rye and ryegrass an increased allergen content and higher IgE activity was measured, while a decrease amount of allergen was detected in timothy grass [20], [21], [63]. Studies on birch trees from urban and rural areas demonstrated no differences in the allergen content of the major birch allergen (Bet v 1, 2, 3 and 4), but showed higher chemotactic activity on human neutrophil granulocytes for the urban samples, leading to the conclusion that greater allergenicity depends on more than just the allergen content [14]. Recent results for ragweed pollen sampled along high-traffic roads showed partially higher IgE activity against Amb a 1, 2, 6 and 10. However, no correlation to the measured ozone concentration in the different areas and the pollen allergenicity was obvious [64].

Our data on *Ambrosia* plants that were fumigated with elevated ozone over the entire vegetation period support the idea that ozone exhibits an influence on stress-induced transcripts, cell wall components and wax but no direct influence on the Amb a 1 allergen content as tested by ELISA. As it is known from the literature that pollen from several plant species grown in urban habitats show higher allergenicity despite not showing higher allergen or protein content [14], [62], it is still possible that ozone-fumigated ragweed pollen shows higher allergenic potential. In ozone-treated grass pollen an increase of damaged grain and a release of allergen containing cytoplasmic granules was observed [65]. As we did not see an increase of damaged pollen by SEM, an ozone-induced release of such kind of particles is unlikely. Allergenic potential then, has to be tested in a mouse model or by prick tests.

## Supporting Information

Figure S1 **Light conditions (a) and mean temperature/relative humidity (b) in the chambers.**
(PDF)

Figure S2 **Workflow of the** ***Ambrosia*** **transcriptome analysis for defining common/different expressed genes in ragweed pollen; ozone treatment (80 ppb ozone), control (40 ppb ozone).**
(PDF)

Figure S3 **ATR-FTIR spectra of lipid/pectinC mixtures at two different weight ratios.** **a)** absorption spectra of the lipid pectin C mixtures in a range of wavenumbers between 900–3050 cm^−1^. Lipid/pectin mixture 1/500 w/w is given in black; the mixture 1/1000 w/w is given in grey. **b)** difference absorption spectra of lipid/pectinC mixture (1/500 w/w) minus lipid/pectinC (1/1000 w/w).
(PDF)

Figure S4 **Typical RP-HPLC diagram of water-soluble and methanol-extractable metabolites (a–f) and accumulation of individual metabolites (g–j) from ragweed pollen.**
(PDF)

Figure S5 **Sequence length distribution of the original 454-reads (a); of contigs assembled with Newbler 2.5 (b).**
(PDF)

Figure S6 **Comparison of high and low expressed transcripts, qRT-PCR** ***vs.*** **RPKM.**
(PDF)

Figure S7 **Representative immunoblot of expression of Amb a 1 allergen in crude** ***Ambrosia*** **pollen extracts.**
(PDF)

Table S1 **Sequence statistics of the** ***Ambrosia*** **transcriptome sequencing data set.**
(PDF)

Table S2 **Ensemble** ***Ambrosia*** **transcriptome assembly.**
(PDF)

Table S3 **Mapping of 454-Reads to the ensemble** ***Ambrosia*** **transcriptome assembly.**
(PDF)

Table S4 **Identification of environmental-specific** ***Ambrosia*** **transcripts using different RPKM thresholds to filter low-abundant transcripts.**
(PDF)

Table S5 **Distribution of determined RPKM values.**
(PDF)

Table S6 **A detailed description including all tagged genes is provided as an Excel file.** Including gene descriptions and RPKM values.
(XLSX)

Table S7 ***Arabidopsis*** **genes sorted to different BIN codes.**
(XLSX)

Table S8 ***Ambrosia artemisiifolia*** **hits matched to known allergen sequences.**
(XLSX)
