# Supplementary material for: Molecular and Immunological Characterization of Ragweed (Ambrosia artemisiifolia L.) Pollen after Exposure of the Plants to Elevated Ozone over a Whole Growing Season
Source: PLoS One. 2013 Apr 18;8(4):e61518. doi: 10.1371/journal.pone.0061518 (PMC3630196; doi:10.1371/journal.pone.0061518)
Supplement: Figure S1 — Light conditions (a) and mean temperature/relative humidity (b) in the chambers. (PDF) [file pone.0061518.s001.pdf]

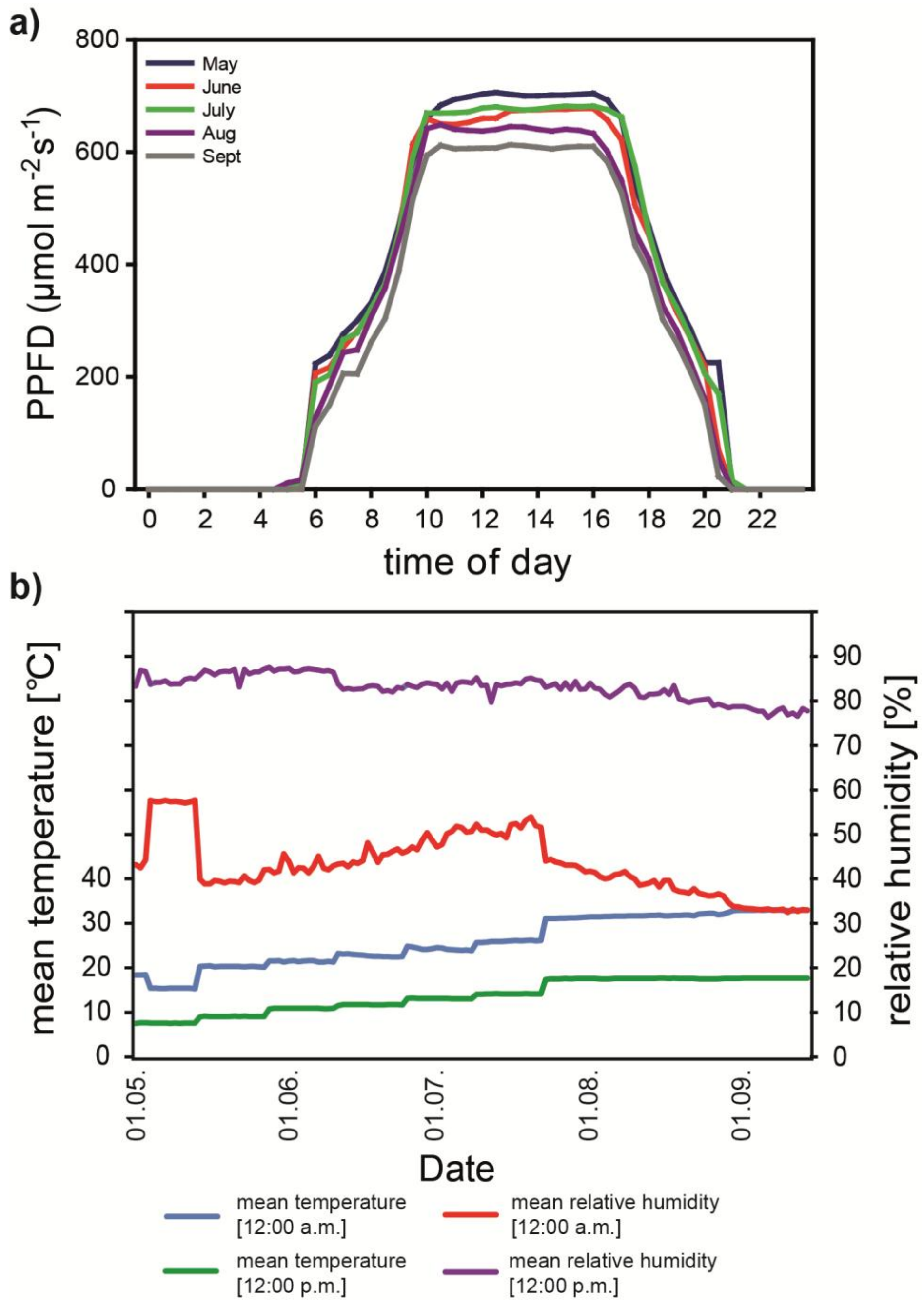

Figure S1. Light conditions and mean temperature / relative humidity in the exposure chambers. Graph **(a)** shows the lightening time and light intensity representative for five different time point of the cultivation season. PPFD = Photosynthetic Photon Flux Density. In graph **(b)** the mean temperature and relative humidity at 12:00 a.m. and 12:00 p.m., respectively are given for the whole growth period.
