# Supplementary material for: Molecular and Immunological Characterization of Ragweed (Ambrosia artemisiifolia L.) Pollen after Exposure of the Plants to Elevated Ozone over a Whole Growing Season
Source: PLoS One. 2013 Apr 18;8(4):e61518. doi: 10.1371/journal.pone.0061518 (PMC3630196; doi:10.1371/journal.pone.0061518)
Supplement: Figure S2 — Workflow of the Ambrosia transcriptome analysis for defining common/different expressed genes in ragweed pollen; ozone treatment (80 ppb ozone), control (40 ppb ozone). (PDF) [file pone.0061518.s002.pdf]

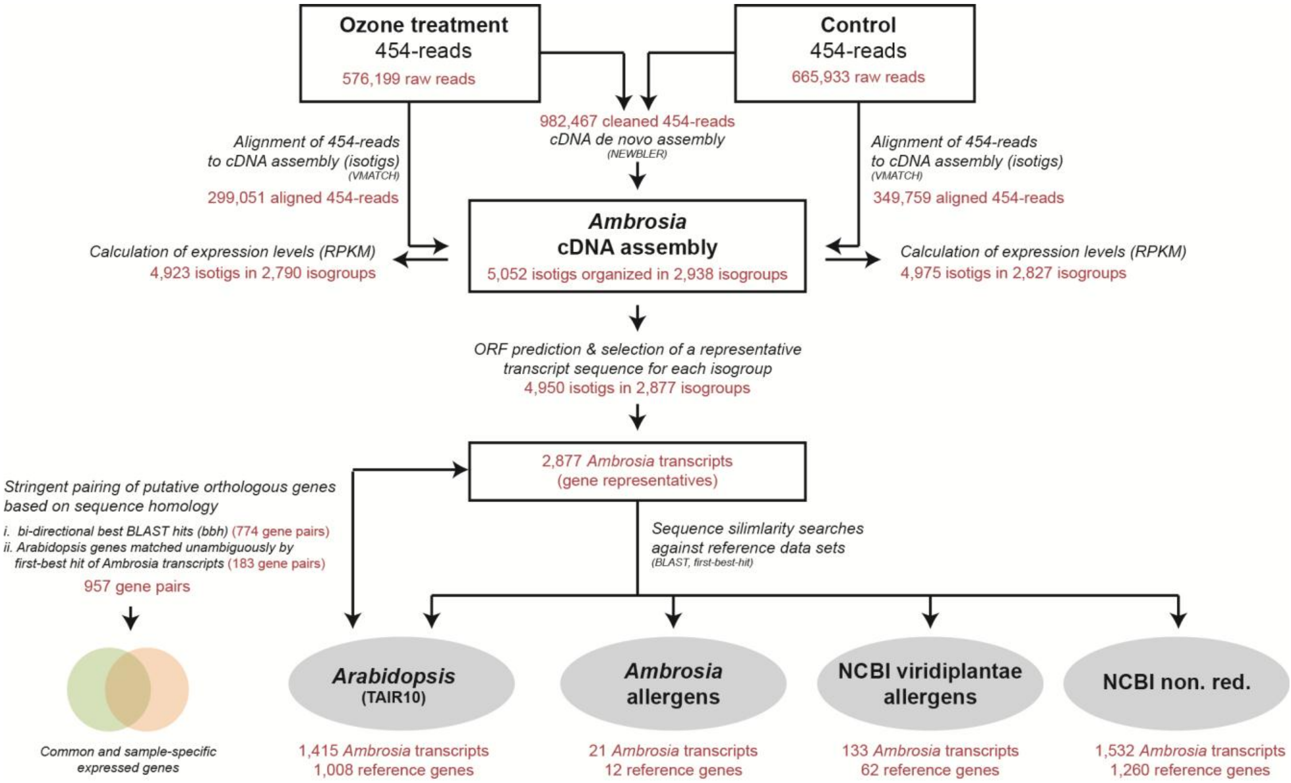

Figure S2. Workflow of the *Ambrosia* transcriptome analysis. *Ambrosia* plants were cultivated under 80 ppb ozone (ozone group) and 40 ppb ozone (control group) in the air, respectively and pollen transcriptomes were sequenced using the 454 technology. A comprehensive *de novo* assembly of the *Ambrosia* transcriptome was created based on an ensemble data set incorporating raw sequencing reads of both groups and transcript expression levels were measured for the assembled transcripts under both conditions. Therefore, 454-reads were aligned against the assembled isotigs. Based on the number of mapped reads, expression quantified in reads per kilobase of exon model per million mapped reads (RPKM) (Mortazavi et al, 2008). The assembled transcriptome was subjected to functional annotation and, thus, protein sequences were predicted for all transcripts. Alternative splice variants were removed from the data set keeping the longest peptide sequence predictions. Sequence similarities were calculated against the *Arabidopsis* gene set, *Ambrosia* candidate allergens, plant allergens downloaded from NCBI and the entire NCBI non-redundant protein database, respectively. Additionally, *Ambrosia* transcripts were stringently paired with the *Arabidopsis* genes via best-bidirectional BLAST hit and unambiguous first-best-hit criteria.
