# Supplementary material for: Molecular and Immunological Characterization of Ragweed (Ambrosia artemisiifolia L.) Pollen after Exposure of the Plants to Elevated Ozone over a Whole Growing Season
Source: PLoS One. 2013 Apr 18;8(4):e61518. doi: 10.1371/journal.pone.0061518 (PMC3630196; doi:10.1371/journal.pone.0061518)
Supplement: Figure S3 — ATR-FTIR spectra of lipid/pectinC mixtures at two different weight ratios. a) absorption spectra of the lipid pectin C mixtures in a range of wavenumbers between 900–3050 cm−1. Lipid/pectin mixture 1/500 w/w is given in black; the mixture 1/1000 w/w is given in grey. b) difference absorption spectra of lipid/pectinC mixture (1/500 w/w) minus lipid/pectinC (1/1000 w/w). (PDF) [file pone.0061518.s003.pdf]

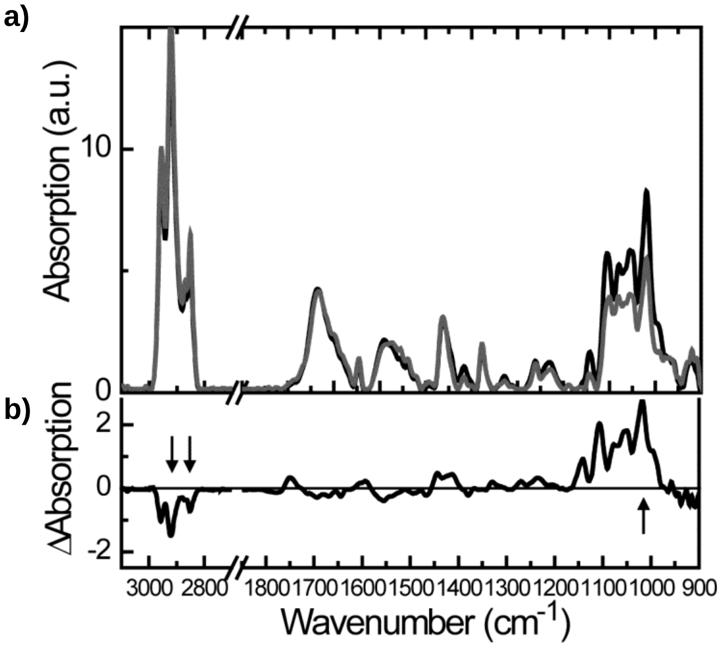


**Figure S3.** ATR-FTIR spectra of lipid/pectin mixtures at two different weight ratios. **a)** shows the absorption spectra of the lipid pectin C mixtures in a range of wavenumbers between 900-3050 $cm^{-1}$. Lipid/pectin mixture 1/500 w/w is given in black, the mixture 1/1000 w/w is given in grey. **b)** shows the difference absorption spectra of lipid/pectinC mixture (1/500 w/w) minus lipid/pectinC (1/1000 w/w). Note, that doubling the pectin C relative to the lipids produces a difference spectrum with an absorption minimum at ~2924 and ~2854 $cm^{-1}$ (major absorption bands of lipid acyl chain) and an absorption maximum at ~1022 $cm^{-1}$ (major absorption band of secondary alcohols of pectin).
