# Supplementary material for: Molecular and Immunological Characterization of Ragweed (Ambrosia artemisiifolia L.) Pollen after Exposure of the Plants to Elevated Ozone over a Whole Growing Season
Source: PLoS One. 2013 Apr 18;8(4):e61518. doi: 10.1371/journal.pone.0061518 (PMC3630196; doi:10.1371/journal.pone.0061518)
Supplement: Figure S4 — Typical RP-HPLC diagram of water-soluble and methanol-extractable metabolites (a–f) and accumulation of individual metabolites (g–j) from ragweed pollen. (PDF) [file pone.0061518.s004.pdf]

a)

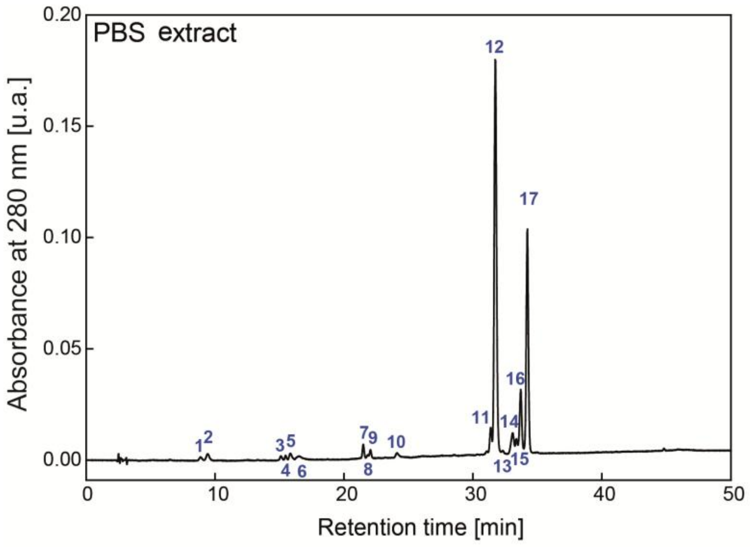

b)

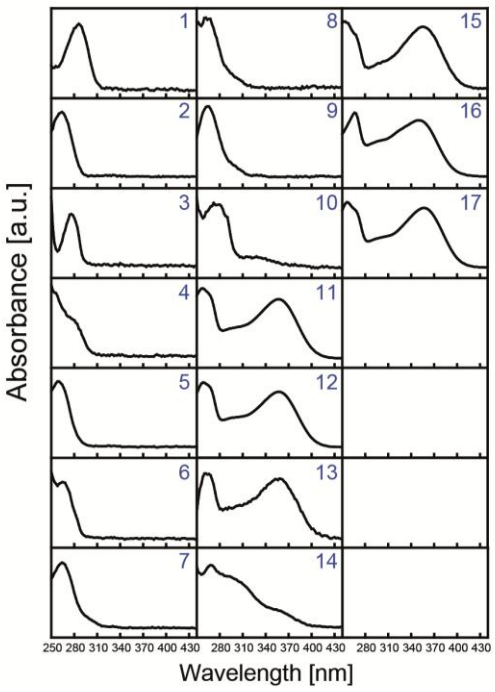

c)

#	Time	Maxima	Preliminary assignment
**1**	8.8	285	
**2**	9.3	263	
**3**	14.8	274	
**4**	15.1	255-277	
**5**	15.4	258	
**6**	16.2	264	
**7**	21.4	263	
**8**	21.7	260-266	
**9**	22.0	263	
**10**	24.0	272-278-289	
**11**	31.4	256-356	Quercetin 3-O-glycoside
**12**	31.7	257-356	Quercetin 3-O-glycoside
**13**	32.3	261-264-356	Quercetin 3-O-glycoside
**14**	33.2	267	
**15**	33.4	252-266-354	Quercetin 3-O-glycoside
**16**	33.8	265-348	Kaempferol 3-O-glycoside
**17**	34.3	256-356	Quercetin 3-O-glycoside

d)

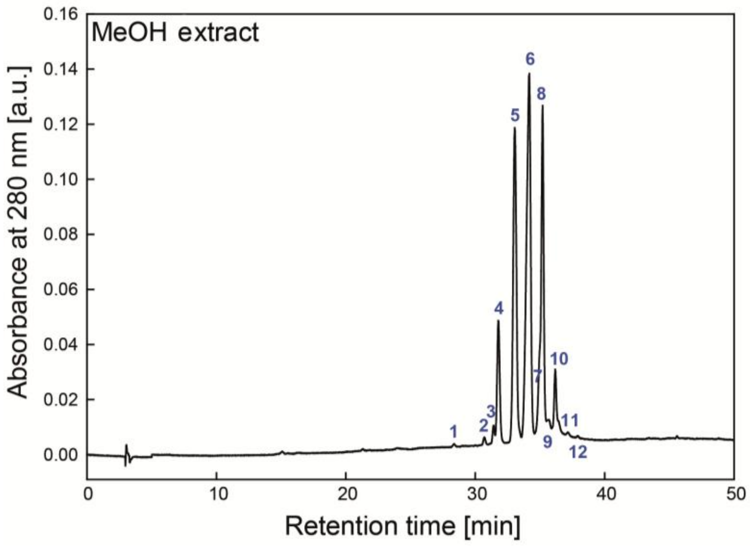

e)

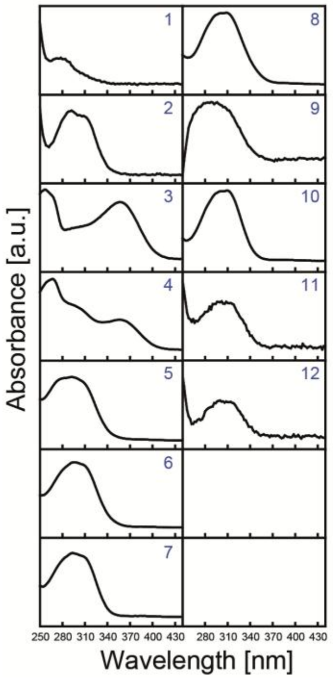

f)

#	Time	Maxima	Preliminary assignment
**1**	28.4	276	
**2**	30.7	290-306	
**3**	31.4	255-355	= PBS 11
**4**	31.8	265-300-355	= PBS 12 + ?
**5**	33.1	290	
**6**	34.2	295	
**7**	35.0	292	
**8**	35.2	308	
**9**	35.6	290	
**10**	36.2	308	
**11**	37.1	308	
**12**	37.9	300-312	

g)

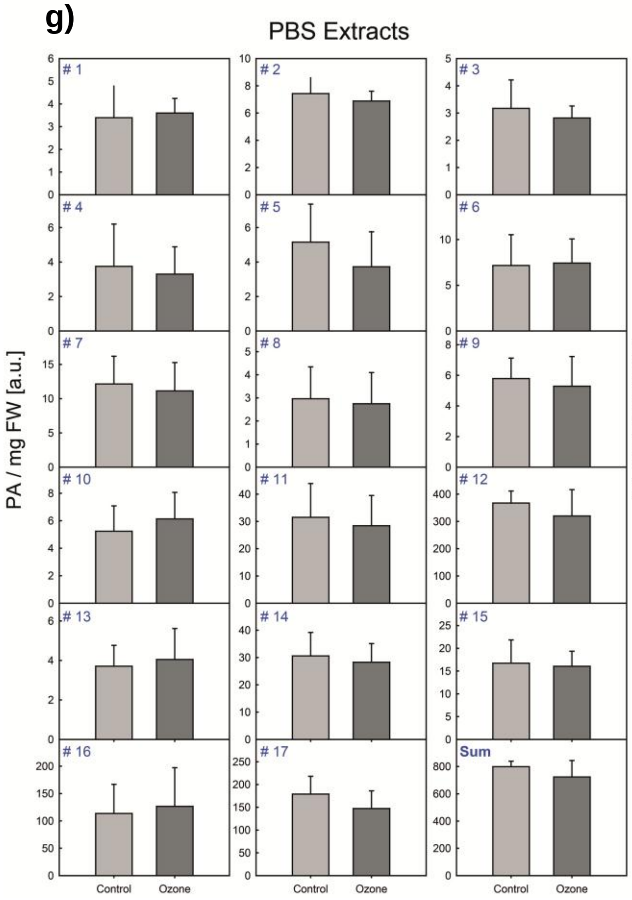
PBS Extracts
PA / mg FW [a.u.]
1
2
3
4
5
6
7
8
9
10
11
12
13
14
15
16
17
Sum
Control
Ozone

h)

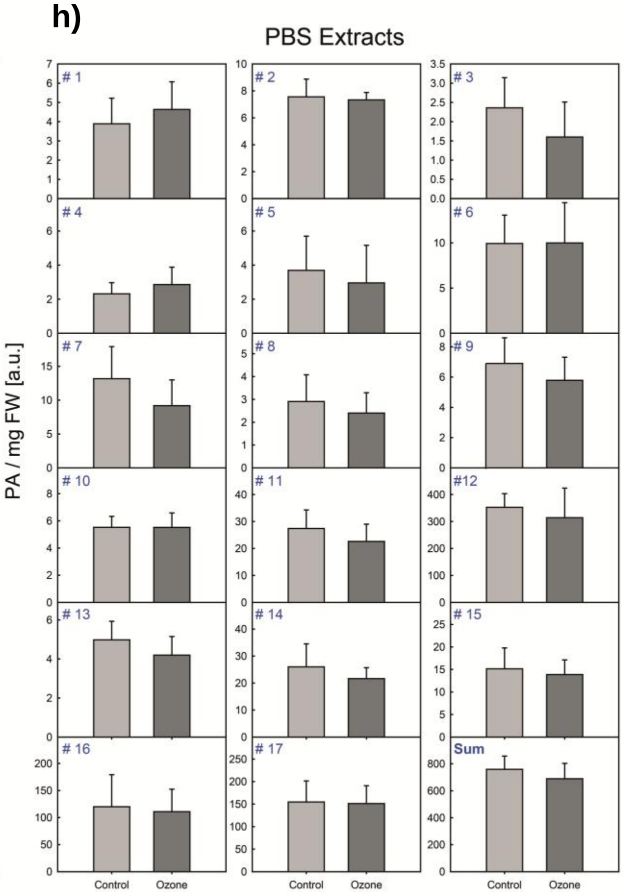
PBS Extracts
PA / mg FW [a.u.]
1
2
3
4
5
6
7
8
9
10
11
#12
13
14
15
16
17
Sum
Control
Ozone

i)

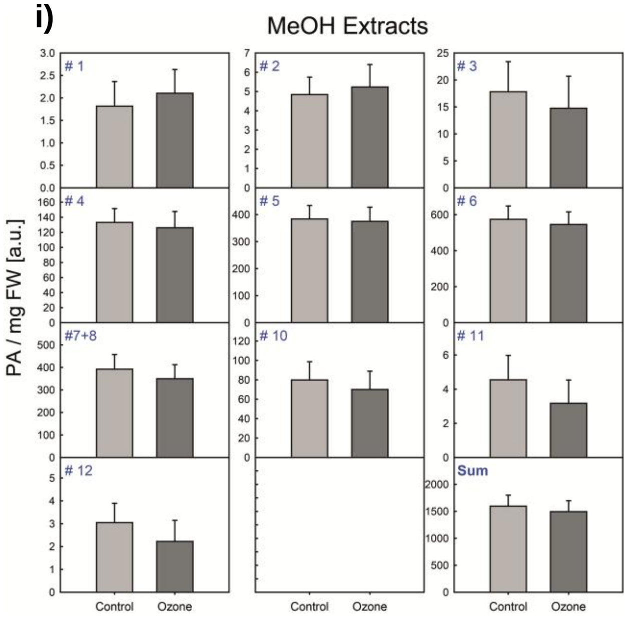
MeOH Extracts
PA / mg FW [a.u.]
1
2
3
4
5
6
#7+8
10
11
12
Sum
Control
Ozone

j)

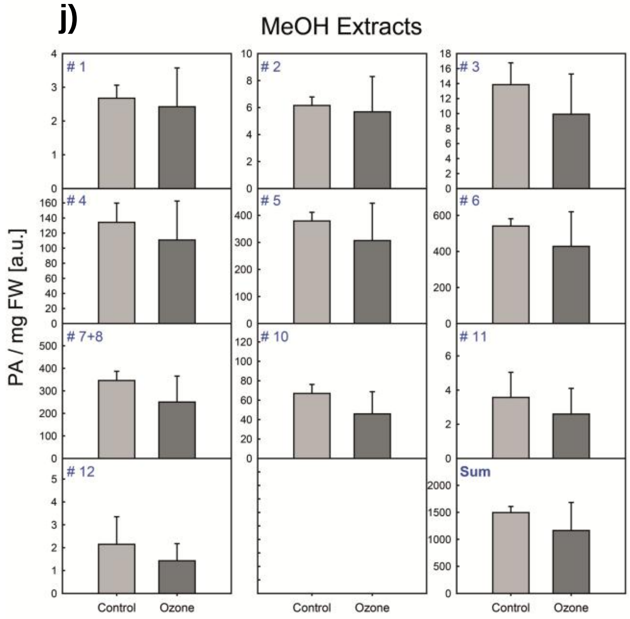
MeOH Extracts
PA / mg FW [a.u.]
1
2
3
4
5
6
7+8
10
11
12
Sum
Control
Ozone

Figure S4. **(a)** shows a typical HPLC run of a PBS extract from *Ambrosia* pollen at an absorbance of 280 nm, resulting in 17 distinct peaks. **(b)** represents the diode array spectra of the respective peaks between 250 and 430 nm. **(c)** indicates the elution times and the maxima of the corresponding peaks. For the peaks #11-13 and #15-17 preliminary structural assignments are given. **(d)** gives a typical HPLC run of a methanol extract, following the PBS extraction, from *Ambrosia* pollen at an absorbancy of 280 nm. The HPLC run results in 12 distinct peaks. Diode array spectra of the respective peaks between 250 and 430 nm are given in **(e)**. **(f)** indicates the corresponding retention time and the peak maxima for each single peak stated in figure part (d). Peak # 3 corresponds to the peak 11 from the PBS extraction. Peak # 4 contains in part the compounds related to peak 12 of the PBS extract besides an unknown compound.

On the basis of peak area obtained at 280 nm, the PBS and methanol extractable metabolites from *Ambrosia artemisiifolia* pollen are given. **(g)** represents the PBS soluble fraction from pollen sampled on August 23, and **(h)** the respective ones sampled on August 30. **(i)** and **(j)** represent the peak areas of methanol extracted metabolites after PBS extraction sampled on August 23 and August 30, respectively. PBS = phosphate buffer saline; PA = peak area; FW = fresh weight.
