# Supplementary material for: Molecular and Immunological Characterization of Ragweed (Ambrosia artemisiifolia L.) Pollen after Exposure of the Plants to Elevated Ozone over a Whole Growing Season
Source: PLoS One. 2013 Apr 18;8(4):e61518. doi: 10.1371/journal.pone.0061518 (PMC3630196; doi:10.1371/journal.pone.0061518)
Supplement: Figure S5 — Sequence length distribution of the original 454-reads (a); of contigs assembled with Newbler 2.5 (b). (PDF) [file pone.0061518.s005.pdf]

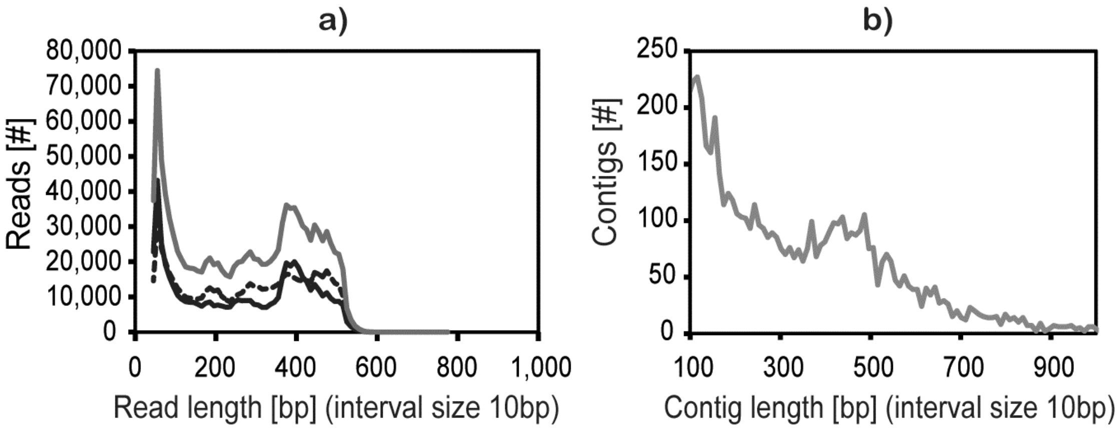

Figure S5. (a) Distribution of the length of the original 454-reads. **(b)** Sequence length distribution of contigs assembled with Newbler 2.5, removing contigs with sequence length <100 bp; (**—** = ozone.454-reads, **—** = control.454-reads, — = ensemble
