# Supplementary material for: Molecular and Immunological Characterization of Ragweed (Ambrosia artemisiifolia L.) Pollen after Exposure of the Plants to Elevated Ozone over a Whole Growing Season
Source: PLoS One. 2013 Apr 18;8(4):e61518. doi: 10.1371/journal.pone.0061518 (PMC3630196; doi:10.1371/journal.pone.0061518)
Supplement: Figure S6 — Comparison of high and low expressed transcripts, qRT-PCR vs. RPKM. (PDF) [file pone.0061518.s006.pdf]

a)

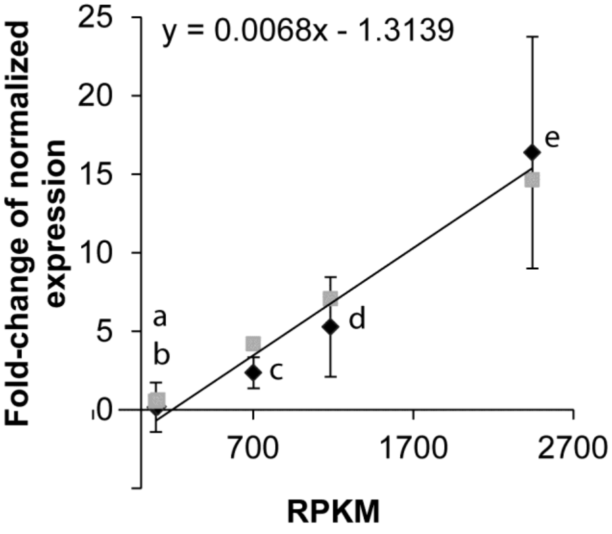

b)

Representative isoform	Corresponding dot in figure	Name	Sequence
**Isotig01227**		Tubulin Fwd	TGCAGAGGGCTGTTTGCATGA
**Isotig01227**		Tubulin Rev	ACCCACGTACCAGTGAACAAAAG
**Isotig02972**	a	Art v 2 Fwd	GGCTGATCAAAGGAAGGACTGCAA
**Isotig02972**	a	Art v 2 Rev	TGCACCGCGTATGAACCATTGA
**Isotig03100**	b	Amb a 10 Fwd	GACGAGCTATTCGCCATCCTGAAA
**Isotig03100**	b	Amb a 10 Rev	GCTAATGAAACCATCGCCATCTGC
**Contig32361**	c	Amba 8 2 Fwd	GAATTCGATGCAGCTGGTACCCTT
**Contig32361**	c	Amb a 8.2 Rev	GGGCTCTTCGTAAATGCCAAACAC
**Isotig03734**	d	Clathrin Fwd	AGGCGTTCATTCGCGCTTACTA
**Isotig03734**	d	Clathrin Rev	ATCCTGGAGATCCTGCAACCAAAC
**Isotig02293**	e	02293 Fwd	AGCTCACTTGGATGCTCACCTTCT
**Isotig02293**	e	02293 Rev	CCATGGGCAAGGTGGAAGTTGTA

Figure S6. Comparison of high and low expressed transcripts, qRT-PCR *vs.* RPKM. **a)** Fold-change of tubulin normalized expression data are plotted against high and low RPKM-values of transcripts from control plants (black) (n=3). RPKM-values normalized to tubulin are shown in grey. Pearson-correlation r = 0.984. **b)** Corresponding isoforms with primer sequences are indicated.
