# Supplementary material for: Molecular and Immunological Characterization of Ragweed (Ambrosia artemisiifolia L.) Pollen after Exposure of the Plants to Elevated Ozone over a Whole Growing Season
Source: PLoS One. 2013 Apr 18;8(4):e61518. doi: 10.1371/journal.pone.0061518 (PMC3630196; doi:10.1371/journal.pone.0061518)
Supplement: Figure S7 — Representative immunoblot of expression of Amb a 1 allergen in crude Ambrosia pollen extracts. (PDF) [file pone.0061518.s007.pdf]

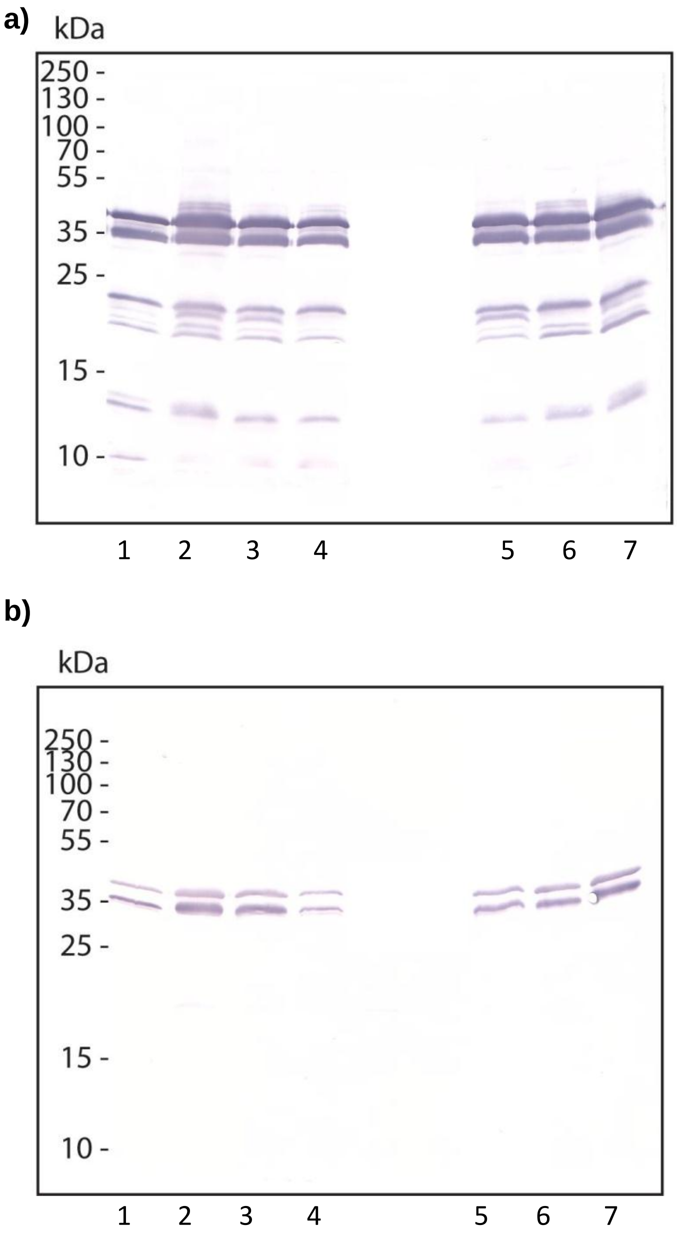

Figure S7. Representative immunoblot of expression of Amb a 1 allergen in crude *Ambrosia* pollen extracts. 1: Allergon extract, 2: ozone (120 ppb), 3: control, 4: ozone (120 ppb), 5: control, 6: ozone (80 ppb), 7: control. 10 µg of total protein per lane. Proteins were separated on a SDS-PAGE, electroblotted onto nitrocellulose membranes. After incubation with specific 1st antibodies, bound antibodies were detected with alkaline phosphatase-conjugated to goat anti rabbit IgG and rabbit anti mouse IgG, IgM, respectively. Labelling was detected using the NBT/bromo-chloro-indolyl phosphate substrate. **a)** 1st AB: polyclonal rabbit anti Amb a 1 serum, 2nd AB: AP-conjugated goat anti rabbit IgG. **b)** 1st AB: murine mAB anti Amb a 1 A39, 2nd AB: AP-conjugated rabbit anti mouse IgG, IgM.
