# Supplementary material for: Molecular and Immunological Characterization of Ragweed (Ambrosia artemisiifolia L.) Pollen after Exposure of the Plants to Elevated Ozone over a Whole Growing Season
Source: PLoS One. 2013 Apr 18;8(4):e61518. doi: 10.1371/journal.pone.0061518 (PMC3630196; doi:10.1371/journal.pone.0061518)
Supplement: Table S1 — Sequence statistics of the Ambrosia transcriptome sequencing data set. (PDF) [file pone.0061518.s008.pdf]

Table S1. Sequence statistics of the *Ambrosia* transcriptome sequencing data sets

	Ozone 454-reads	Control 454-reads	Ensemble
**Reads < 100bp (removed) [#]**	138,818	120,847	259,665
**Cleaned reads [#]**	437,381	545,086	982,467
**Cleaned total Sequence [bp]**	144,590,356	179,214,471	323,804,827
**Read Length**			
**Minimal [bp]**	100	100	100
**Maximal [bp]**	772	677	772
**Mean [bp]**	330.58	328.78	329.58
**GC-Content [%]**	43.35	43.78	43.56
**N's [bp] / [%]**	17,616 / 0.01	63,674 / 0.04	81,290 / 0.03
