# Supplementary material for: Molecular and Immunological Characterization of Ragweed (Ambrosia artemisiifolia L.) Pollen after Exposure of the Plants to Elevated Ozone over a Whole Growing Season
Source: PLoS One. 2013 Apr 18;8(4):e61518. doi: 10.1371/journal.pone.0061518 (PMC3630196; doi:10.1371/journal.pone.0061518)
Supplement: Table S2 — Ensemble Ambrosia transcriptome assembly. (PDF) [file pone.0061518.s009.pdf]

Table S2. Ensemble *Ambrosia* transcriptome assembly

**Contigs (≥ 100bp) [#]**	5,720
**Contig Sequence [bp]**	2,122,363
**Contig Length**	
**Minimal [bp]**	100
**Maximal [bp]**	2,166
**Mean [bp]**	371.04
**Singletons [#]**	49,729
**Singleton Sequence [bp]**	13,696,252
**Singleton Length**	
**Minimal [bp]**	100
**Maximal [bp]**	772
**Mean [bp]**	275.42
**Isogroups [#]**	2,938
**with isotigs that have ORFs**	2,877
**without isotigs that have ORFs**	61
**Isotigs [#]**	5,052
**with ORF (≥100nt)**	4,950
**without ORF (≥100nt)**	102
**Isotig Sequence [bp]**	3,147,787
**Isotig Length**	
**Minimal [bp]**	112
**Maximal [bp]**	5,748
**Mean [bp]**	623.08
