# Supplementary material for: Molecular and Immunological Characterization of Ragweed (Ambrosia artemisiifolia L.) Pollen after Exposure of the Plants to Elevated Ozone over a Whole Growing Season
Source: PLoS One. 2013 Apr 18;8(4):e61518. doi: 10.1371/journal.pone.0061518 (PMC3630196; doi:10.1371/journal.pone.0061518)
Supplement: Table S3 — Mapping of 454-Reads to the ensemble Ambrosia transcriptome assembly. (PDF) [file pone.0061518.s010.pdf]

Table S3. Mapping of 454-reads to the ensemble *Ambrosia* transcriptome assembly

	Ozone 454-reads	Control 454-reads
**Mapped 454-Reads**	299,051 (52%)	349,759 (53%)
**Mapped to isotig(s) of the same isogroup**	294,173 (98%)	343,292 (98%)
**Mapped to isotig(s) of different isogroups**	4,878 (2%)	6,467 (2%)
**Mapped to unique isotig**	244,513 (82%)	281,841 (81%)
**Mapped to multiple isotigs**	54,538 (18%)	67,918 (19%)
**Total isogroups with isotigs matched by 454-reads**	2,855 (97%)	2,884 (98%)
**Total of all assembled isoforms matched by 454-reads**	4,923 (97%)	4,975 (98%)
**Total matched isogroup representatives by 454-reads**	2,790 (97%)	2,827 (98%)
