# Supplementary material for: Molecular and Immunological Characterization of Ragweed (Ambrosia artemisiifolia L.) Pollen after Exposure of the Plants to Elevated Ozone over a Whole Growing Season
Source: PLoS One. 2013 Apr 18;8(4):e61518. doi: 10.1371/journal.pone.0061518 (PMC3630196; doi:10.1371/journal.pone.0061518)
Supplement: Table S4 — Identification of environmental-specific Ambrosia transcripts using different RPKM thresholds to filter low-abundant transcripts. (PDF) [file pone.0061518.s011.pdf]

Table S4. Identification of environmental-specific *Ambrosia* transcripts using different RPKM thresholds to filter low-abundant transcripts

RPKM	Ozone specific	Control specific	Ozone+Control	None
**≥1**	50	87	2,740	0
**≥2**	81	87	2,739	0
**≥3**	54	88	2,735	0
**≥4**	60	92	2,724	1
**≥5**	71	102	2,703	1
**≥6**	89	120	2,666	2
**≥7**	116	150	2,607	4
**≥8**	139	186	2,547	5
**≥9**	154	207	2,509	7
**≥10**	167	218	2,481	11
