# Supplementary material for: Molecular and Immunological Characterization of Ragweed (Ambrosia artemisiifolia L.) Pollen after Exposure of the Plants to Elevated Ozone over a Whole Growing Season
Source: PLoS One. 2013 Apr 18;8(4):e61518. doi: 10.1371/journal.pone.0061518 (PMC3630196; doi:10.1371/journal.pone.0061518)
Supplement: Table S5 — Distribution of determined RPKM values. (PDF) [file pone.0061518.s012.pdf]

Table S5. Distribution of determined RPKM values

	Ozone specific	Control specific	Ozone+Control
**Minimum**	0.0	0.0	0.0
**Maximum**	205,090.1	154,588.4	205,090.1
**Mean**	620.1	610.4	615.3
**Standard deviation**	5,899.8	5,521.9	5,713.5
**5th Percentile**	6.8	7.9	7.3
**Median**	49.8	48.1	48.9
**95th Percentile**	1,175.9	1,076.1	1,119.5
